# The Availability and Consistency of Dengue Surveillance Data Provided Online by the World Health Organization

**DOI:** 10.1371/journal.pntd.0003511

**Published:** 2015-04-14

**Authors:** Irene Ruberto, Ernesto Marques, Donald S. Burke, Willem G. Van Panhuis

**Affiliations:** 1 Department of Epidemiology, University of Pittsburgh Graduate School of Public Health, Pittsburgh, Pennsylvania, United States of America; 2 Center for Vaccine Research, University of Pittsburgh, Pittsburgh, Pennsylvania, United States of America; Centers for Disease Control and Prevention, UNITED STATES

## Abstract

**Background:**

The use of high quality disease surveillance data has become increasingly important for public health action against new threats. In response, countries have developed a wide range of disease surveillance systems enabled by technological advancements. The heterogeneity and complexity of country data systems have caused a growing need for international organizations such as the World Health Organization (WHO) to coordinate the standardization, integration, and dissemination of country disease data at the global level for research and policy. The availability and consistency of currently available disease surveillance data at the global level are unclear. We investigated this for dengue surveillance data provided online by the WHO.

**Methods and Findings:**

We extracted all dengue surveillance data provided online by WHO Headquarters and Regional Offices (RO’s). We assessed the availability and consistency of these data by comparing indicators within and between sources. We also assessed the consistency of dengue data provided online by two example countries (Brazil and Indonesia). Data were available from WHO for 100 countries since 1955 representing a total of 23 million dengue cases and 82 thousand deaths ever reported to WHO. The availability of data on DengueNet and some RO’s declined dramatically after 2005. Consistency was lacking between sources (84% across all indicators representing a discrepancy of almost half a million cases). Within sources, data at high spatial resolution were often incomplete.

**Conclusions:**

The decline of publicly available, integrated dengue surveillance data at the global level will limit opportunities for research, policy, and advocacy. A new financial and operational framework will be necessary for innovation and for the continued availability of integrated country disease data at the global level.

## Author Summary

The use of high quality data and information has become essential for public health agencies to monitor and protect population health. Technological advancement has enabled the development of sophisticated disease surveillance systems by many countries. Increasingly, countries are making surveillance data publicly available to their constituencies. A key role of international agencies such as the World Health Organization is the integration and curation of country data at the global level. Because it can be confusing to navigate the current online disease data landscape, we assessed the availability and consistency of online available surveillance data for dengue provided by the World Health Organization and two example countries (Brazil and Indonesia). We found that data availability declined substantially after 2005 and that consistency between sources was limited to 84%, representing a discrepancy of half a million cases. These limitations reduce opportunities for the efficient use of country data to counter public health threats. A new financial and operational model is needed to advance the use of disease data at the global level. Industry and academic partners need to step up to support this mandate.

## Introduction

Threats to public health around the world have become increasingly complex and the importance of high quality disease surveillance for preparedness and disease control will continue to grow [1]. Scientific progress and global cooperation against emerging threats will depend on the availability and sharing of disease surveillance data between countries. Global health and funding agencies emphasized this in an appeal for greater availability and use of data for global health [2,3]. Formally, the 2005 International Health Regulations require the use and sharing of data in response to new threats [4,5]. The central role of the World Health Organization (WHO) in global disease surveillance and data dissemination has been stated in World Health Assembly resolutions for specific diseases [6].

The WHO has developed various data systems to integrate and disseminate country surveillance data such as the Global Health Observatory [7], the Global TB Database [8], DengueNet [9], RabNet [10] and FluNet [11]. In addition to these global databases, WHO Regional Offices (RO’s) also provide disease surveillance data through their websites to inform member countries on disease patterns and trends in their region. Increasingly, country Ministries of Health post their own disease surveillance data online for their constituency, mostly in the form of epidemiological bulletins but sometimes using sophisticated online data repositories such as those developed by Brazil and Indonesia [12,13]. The public availability of disease surveillance data from various heterogeneous sources provides new opportunities for research, training, and policy making but can also lead to confusion on data discrepancies between sources. Limited information on methodology used at various steps along the data trail from within countries to the global level has further complicated this data landscape. Although it is generally known that surveillance data reported by different agencies may not be identical due to reporting methods and definitions, few studies have quantified the availability and consistency of publicly available disease surveillance data across sources. This information can guide policy makers, scientists, students, and others to use available data more effectively. We used the example of dengue to assess the availability and consistency of surveillance data provided online by WHO. We also provided examples of online data provided by the Ministry of Health of Brazil and Indonesia.

## Methods

We extracted all online dengue surveillance data from WHO (WHO DengueNet [9] and from the websites of the Pan American Health Organization (PAHO) [14], the WHO Southeast Asia Regional Office (SEARO) [15] and the WHO Western Pacific Regional Office (WPRO) [16,17]), and by the Ministries of Health (MOH) of Brazil and Indonesia [12,13]. Brazil and Indonesia were selected as examples because they provided open access to detailed dengue surveillance data online in computer readable format. All available data up to April 12^th^ 2013 were extracted at the highest possible spatiotemporal resolution. To obtain standardized data across these sources, we extracted data for all ages and for both genders combined. We did not extract serotype specific data because these were minimally available. We standardized indicators reported by different sources across spatial and temporal scales and also harmonized country names using the United Nations ISO country name standard (ISO 3166) [18]. We assessed the availability of dengue data from each source and also measured data consistency between and within sources. We defined consistency between sources as the percent agreement of data reported for overlapping countries and time periods. We defined consistency within a source by the percent agreement of indicators that were recomputed by us from data within the source and the corresponding indicators provided by the same source.

All data used in this study are made publicly available through the University of Pittsburgh Project Tycho online data system (www.tycho.pitt.edu).

## Results

### Data availability

**Fig 1:**
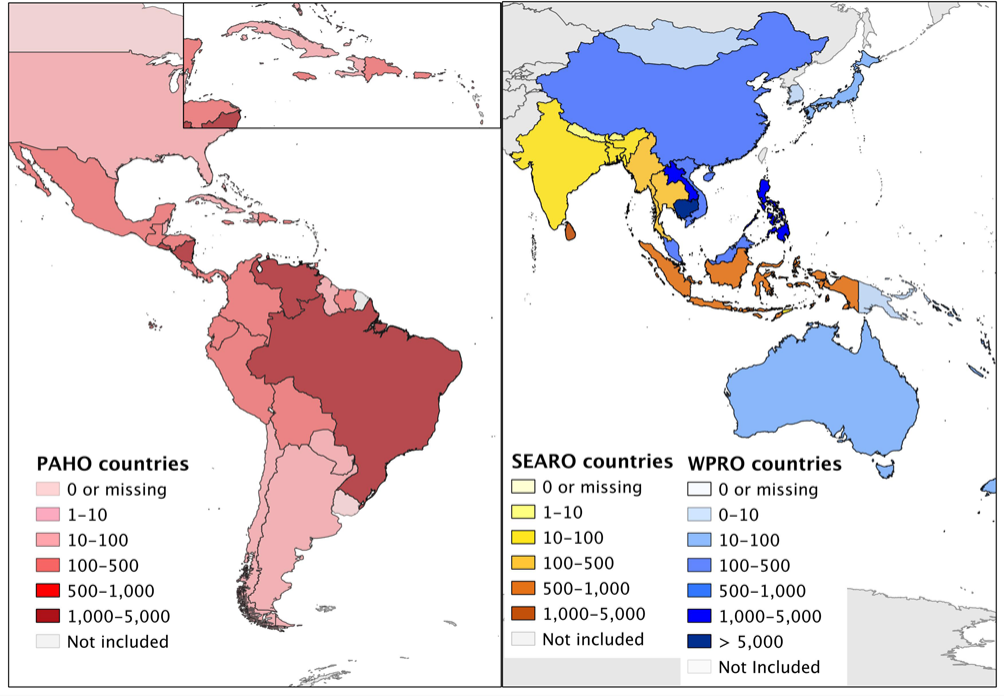
Number of counts per country available from online WHO sources: 1955–2012. A count was defined as a reported value for an indicator, e.g. the reported number of cases for one month and location would be one count. The number of counts available per country (indicated in different colors) was determined by the spatiotemporal resolution of data, the number of indicators reported, and the length of the time period reported.

We extracted a total of 71,460 counts for 100 countries from DengueNet and WHO RO websites (Fig 1). These data represented a total of ~23 million dengue cases and ~82,000 deaths that have been reported to WHO between 1955 and 2012. Of these, ~13 million cases (56%) and ~20,000 deaths (24%) were reported between 2000 and 2012. A total of ~4.6 million cases were reported by WPRO (20%), ~3.2 million (14%) by SEARO, and ~15 million (66%) by PAHO countries (Table 1). The majority of dengue deaths were reported by SEARO (49%) and WPRO (44%).

**Table 1:** The cumulative number (in thousands) of “all” dengue cases, DHF cases and “all” deaths per WHO Region.

	Entire period * (1955–2012)	Last decade^†^ (2000–2012)
**All cases**		
WPRO	4,640 (20%)	1,983 (15%)
SEARO	3,216 (14%)	1,060 (8%)
PAHO	15,001 (66%)	9,974 (77%)
EMRO	0.45 (<0.1%)	0 (0%)
**Total**	**22,858 (100%)**	**13,019 (100%)**
**DHF cases**		
WPRO	159 (31%)	159 (38%)
SEARO	1 (0.2%)	0 (0%)
PAHO	357 (69%)	266 (63%)
EMRO	0 (<0.1%)	0 (0%)
**Total**	**518 (100%)**	**426 (100%)**
**All deaths**		
WPRO	36 (44%)	7 (37%)
SEARO	40 (49%)	8 (39%)
PAHO	6 (7%)	5 (24%)
EMRO	0 (0.5%)	0 (0%)
**Total**	**83 (100%)**	**20 (100%)**

* 1955–2012 for all cases, 1981–2012 for DHF cases, 1956–2012 for all deaths.

^†^ Last decade: 2000–2012.

**Fig 2:**
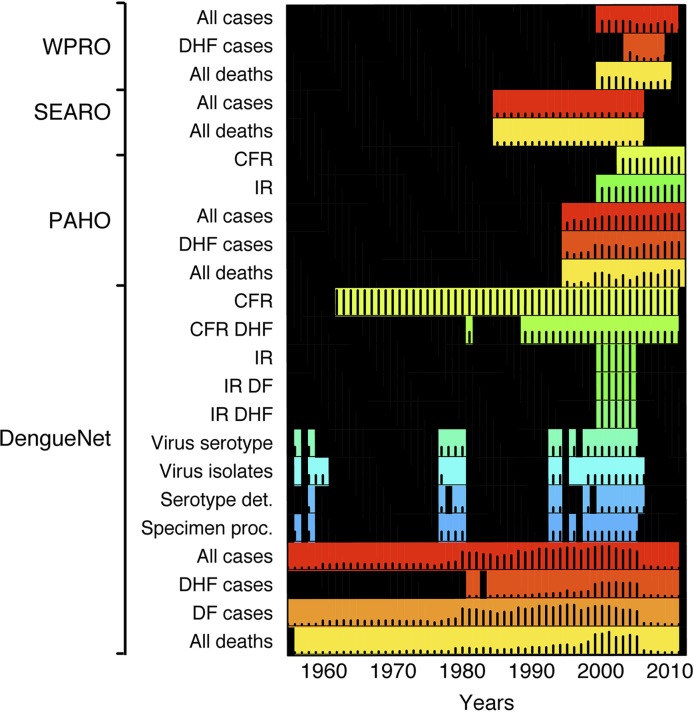
Dengue surveillance indicators available from WHO DengueNet and RO’s (1955–2012). Each indicator was reported by a different number of countries over time (represented by black bars ranging from 0 (bottom) to 83 (top) countries). DHF: Dengue Hemorrhagic Fever, DF: Dengue Fever, All cases: DF+DHF cases; CFR: Case Fatality Rate; IR: Incidence Rate. Different colors represent each unique indicator.

**Fig 3:**
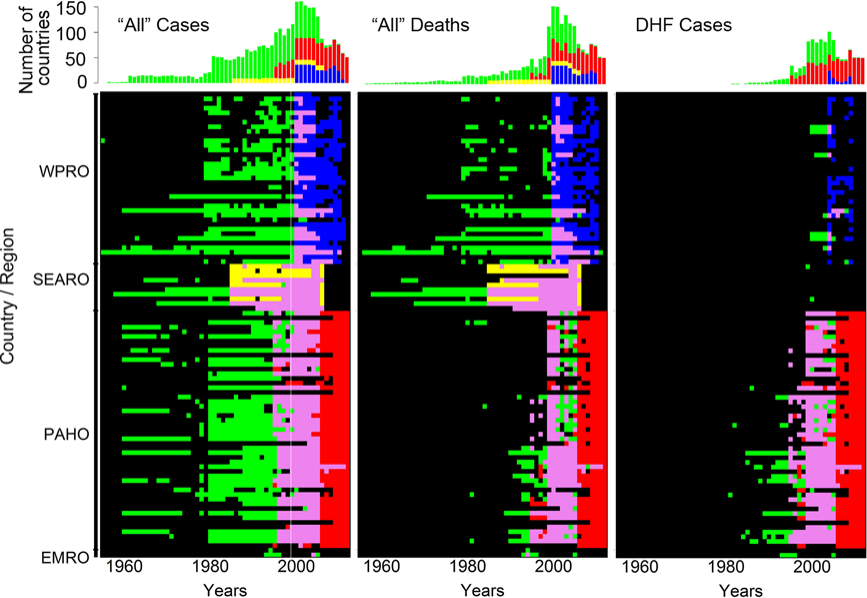
Data availability from WHO DengueNet and Regional Offices per country: 1955–2012. Data availability is shown by coloring vs. black for “all” cases (dengue fever and dengue hemorrhagic fever, DHF), for “all” deaths, and for DHF cases. Data availability per country from DengueNet is indicated in green, from WPRO in blue, from SEARO in yellow, and from PAHO in red. Data points that are available from both DengueNet and a regional office are shown in pink (for more detail see S5A–S5C Fig).

Each source provided counts for a range of different indicators (Fig 2). Data for “all” dengue cases (dengue fever and dengue hemorrhagic fever combined) and “all” dengue deaths were available from DengueNet and all RO’s. Data for DHF cases were predominantly from PAHO, few counts were from WPRO and none from SEARO. Across time, DengueNet provided counts for the longest time period (1955–2011) compared to SEARO (1985–2006), WPRO (2000–2011), and PAHO (1995–2012) (Fig 3 and S1 Fig). Across sources, data for “all” cases were provided for the longest time periods, followed by mortality data. Data for DHF counts were available for the shortest time periods (Fig 3 and S1B Fig). In general, many counts were missing across years and countries.

### Consistency across data sources

We compared data from DengueNet and RO’s to assess consistency across sources (Table 2 and S2 Fig). The overall percent agreement was 83.8% across all indicators. Data from SEARO were the most consistent with a percent agreement of 92.2% and data from WPRO were the least consistent at 72.3%. Data for DHF cases were more consistent compared to the other indicators at 92.4% compared to 76.1% for “all” cases and 89.4% for “all” deaths. DengueNet values for all indicators were generally lower compared to values from RO’s. In total, DengueNet reported 426,808 fewer “all cases”, 17,854 fewer DHF cases, and 245 fewer deaths compared to RO’s (Table 2).

**Table 2:** Consistency between DengueNet and WHO Regional Office data.

Indicator	Source	Percent agreement*	Pairs^†^	Difference^‡^
All cases	PAHO	77.2	219	-398,311
	SEARO	92.5	40	-12,210
	WPRO	64.0	75	-16,287
	**Total**	**76.1**	**334**	**-426,808**
All deaths	PAHO	90.2	164	-67
	SEARO	92.0	50	36
	WPRO	84.0	50	-142
	**Total**	**89.4**	**264**	**-245**
DHF cases	PAHO	92.9	126	-7,854
	WPRO	80.0	5	-10,000
	**Total**	**92.4**	**131**	**-17,854**
All indicators	PAHO	85.3	509	-406,232
	SEARO	92.2	90	-12,246
	WPRO	72.3	130	-26,429
	**Total**	**83.8**	**729**	**- 444,907**

* Percent of pairs with identical values between DengueNet and Regional Office.

^†^ Number of matched pairs excluding missing values.

^‡^ Sum of differences between pairs (DengueNet minus RO).

### Internal consistency of DengueNet data

We recomputed the number of “all” cases for DengueNet from separately reported dengue fever (DF) and DHF cases. We also recomputed the case fatality rate (CFR) for DengueNet from reported cases and deaths. Our recomputed data for “all” cases corresponded with 98.9% of original values and for CFR with 99.5% (Table 3). We also recomputed the annual number of dengue cases at country level from monthly cases at the provincial level (in DengueNet, data were either reported at the country level by year or at the provincial level by month). Our recomputed annual country level data for “all” cases was > 3 million cases lower compared to reported data at that level. The recomputed values for “all” deaths were about 2000 deaths lower compared to reported mortality at country level by year. This discrepancy was likely due to missing data at the lower administrative levels. We found that provincial level data were not available for all calendar months in years before 1997 and after 2004 (S3 Fig). In addition, provincial level data for countries were only available for a median of 3.5% of provinces before 1996 and for 85.7% of provinces after 1996 (using The Second Administrative Level Boundaries data set project (SALB) [19] for the expected number of provinces per country).

**Table 3:** Consistency of data within DengueNet, measured by comparing recomputed with reported indicators.

Indicator	Percent agreement *	Pairs^†^	Difference^‡^
All cases	98.9	446	-27,226
CFR (%)	99.5	789	-0.25
Annual cases at country level	13.3	75	-3,079,617
Annual DF cases at country level	10.9	46	-2,920,397
Annual DHF cases at country level	44.4	36	-3,888
Annual deaths at country level	45.5	55	-2,073

* Percent of pairs with identical values

^†^ Number of matched pairs excluding missing values.

^‡^ Recomputed data minus reported data.

### Data provided online by countries

**Fig 4:**
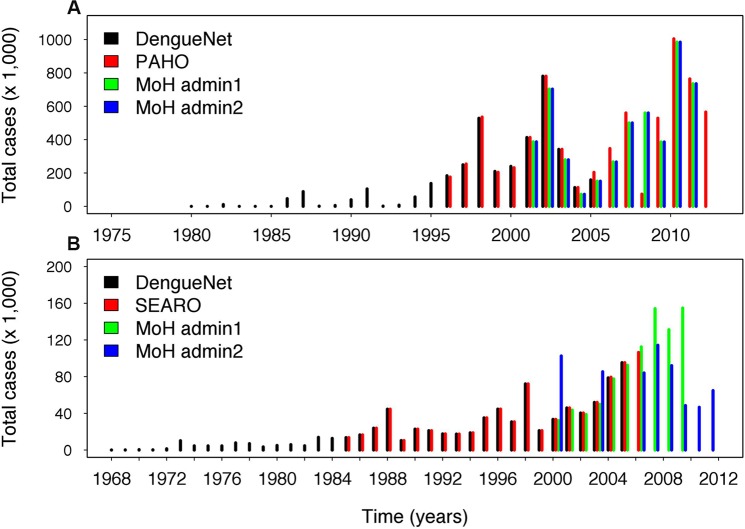
The total number of dengue cases reported for Brazil (A) and Indonesia (B) by different sources. The annual number of “all” cases reported for the entire country from DengueNet, the RO, and the Ministry of Health (MOH). Country level data reported by the MOH was derived from provincial (admin1) and district (admin2) level data provided online.

We also assessed dengue surveillance data provided online by the Ministries of Health of Brazil and Indonesia (Fig 4). Both these countries are dengue endemic and have developed online databases that provide publicly available dengue surveillance data. The annual number of “all” cases reported by the Brazil and Indonesia MOH corresponded to WHO data for most years except 2008 (Brazil) and 2000/2004 (Indonesia). No WHO data were available for Indonesia after 2005. We found discrepancies within the data provided by the MOH of Indonesia for years after 2007. Our recomputed number of cases per year at the country level from reported provincial data (1^st^ administrative level) was higher than country level values recomputed from district data (2^nd^ administrative level). This suggested that data from lower administrative levels were incomplete.

## Discussion

We integrated publicly available online dengue surveillance data from various WHO and country sources to describe the availability and consistency of globally available dengue surveillance data. We found that consistency of overlapping data between DengueNet and WHO Regional Offices was lacking and that data at subnational levels were often incomplete. This incompleteness was difficult to recognize since the absence of data for provinces or districts was not indicated explicitly. DengueNet systematically reported lower values compared to the RO’s. This may be due to a difference in timing of data reports made by countries and a lack of updating DengueNet as countries updated their figures.

DengueNet was created by the WHO Headquarters in 2002 as part of the Global Health Atlas [9,20]. Focal points were appointed and trained in every country to upload standardized reports into the DengueNet repository [21]. This has successfully led to public sharing of dengue data across countries through a central global repository. In addition to DengueNet, RO’s also routinely release dengue surveillance data from member countries through their websites. PAHO and SEARO provide links to surveillance data sheets in PDF format and WPRO has developed an online Health Information and Intelligence Platform (HIIP). The WHO is the only source of integrated disease surveillance data across countries. Numerous studies have used WHO dengue surveillance data to describe trends and patterns of this disease at the global [22,23,24] and regional level [25,26,27]. Despite their role as a core resource for international dengue surveillance data, DengueNet and some RO data have not been regularly updated over the past decade, most likely due to capacity and funding constraints. With the decline of WHO as a central global resource for dengue surveillance data, the data landscape will become increasingly scattered and difficult to navigate. Other agencies or institutes can contribute additional capacity or alternative frameworks for global disease surveillance data may be needed, such as a distributed network instead of centralized databases.

Increasingly, individual countries disseminate their own disease surveillance data online in various formats ranging from epidemiological bulletins to sophisticated databases. This has greatly advanced the availability of disease data at the global level. In 2010 the 63^rd^ World Health Assembly stated that “the WHO urges member states to improve the collection of reliable health information and data and to maximize, where appropriate, their free and unrestricted availability in the public domain” [28]. Country data systems however use a large diversity of surveillance methodology and definitions that often lack detailed documentation. The potential biases and lack of comparability of data across countries are limiting the efficient use of these data. The reporting process of dengue surveillance data from countries to WHO also lacks detailed documentation and may vary across countries. Future research should formally compare country data systems and country vs. WHO data to gain more insight in potential biases of the various sources. A standardized and curated global data system can maximize opportunities for the efficient use of country disease data for science and policy.

Data standardization and curation are essential for a global data system. For example we found that ~16% of country names in DengueNet were different from country names used by the RO’s (S1 Table). Across all WHO sources, ~19% of country names were different from the UN ISO standard for country names [19]. In the absence of up-to-date global platforms for disease surveillance data, alternative data systems have emerged such as Google Dengue and Flu Trends and the HealthMap project that automatically integrate data from search queries or online news items respectively [29,30,31]. Innovative technological solutions and capacity used by these projects should be applied to integrate country disease surveillance data as well to establish a state-of-the-art 21^st^ century global data system. This system can be coordinated by WHO but can be implemented by external institutes that have already created large scale public health data systems such as the Institute of Health Metrics and Evaluation, the Malaria Atlas Project, or Project Tycho.

A new and sustainable framework will be required to ensure that integrated and curated disease surveillance data from countries around the world will continue to be available to stakeholders at all levels. Innovative technology should be used for data integration that minimizes the burden on countries but maximizes data availability and use. Academic and private sector partners should step up to support international agencies with this increasingly complex mandate.

## Supporting Information

S1 Table Country names used by DengueNet, RO’s and corresponding UN ISO names. (PDF)

S1 Fig Data availability from WHO DengueNet and Regional Offices per country: 1955–2012. **(A)** Data for “all” cases (dengue fever and dengue hemorrhagic fever, DHF), **(B)** data for “all” deaths, and **(C)** data for DHF cases. Data availability from DengueNet is indicated in green, from WPRO in blue, from SEARO in yellow, and from PAHO in red. Data points that are available from both DengueNet and a regional office are shown in pink.
(PDF)

S2 Fig Consistency of data from DengueNet and WHO Regional Offices. Data were available from both sources for years between 2000 and 2005 for **(A)** “all” cases, **(B)** “all deaths”, and **(C)** DHF cases.
(PDF)

S3 Fig Completeness of DengueNet data on “all” cases at provincial/monthly resolution across countries by year. Per year, the distribution across countries is shown of the number of calendar months **(A)** and the percent of all provinces **(B)** with data available for “all” cases.
(PDF)
