# Supplementary material for: The Availability and Consistency of Dengue Surveillance Data Provided Online by the World Health Organization
Source: PLoS Negl Trop Dis. 2015 Apr 14;9(4):e0003511. doi: 10.1371/journal.pntd.0003511 (PMC4397048; doi:10.1371/journal.pntd.0003511)
Supplement: S1 Table — (PDF) [file pntd.0003511.s001.pdf]

Table S1. Country names used by DengueNet, Regional Offices and corresponding UN ISO names.

	Country Name		Country WHO Region
**UN ISO 3166 Standard**	**DengueNet**	**Regional Office**	
BRUNEI DARUSSALAM	brunei darussalam	brunei	WPRO
FRENCH POLYNESIA	french polynesia	french polynesia (france)	WPRO
GUADELOUPE	guadeloupe	guadaloupe	PAHO
GUAM	guam	guam (usa)	WPRO
HONG KONG		hongkong (china)<br>hong kong sar (china)	WPRO<br>WPRO
LAO PEOPLE'S DEMOCRATIC REPUBLIC	lao people's democratic republic	lao PDR	WPRO
MACAO		macao sar (china)	WPRO
MICRONESIA, FEDERATED STATES OF	micronesia (federated states of)	micronesia, federated states of	WPRO
NORTHERN MARIANA ISLANDS	northern mariana islands	northern mariana islands, commonwealth of the (usa)	WPRO
PITCAIRN		pitcairn islands<br>pitcairn islands (UK)	WPRO<br>WPRO
SAINT KITTS AND NEVIS	saint kitts and nevis	st. kitts & nevis	PAHO
SAINT LUCIA	saint lucia	st. lucia	PAHO
SAINT VINCENT AND THE GRENADINES	saint vincent and the grenadines	st vincent & the grenadines	PAHO
TIMOR-LESTE	timor-leste	timor leste	SEARO
TRINIDAD AND TOBAGO	trinidad and tobago	trinidad & tobago	PAHO
TURKS AND CAICOS ISLANDS	turks and caicos islands	turks & caicos islands	PAHO
VENEZUELA, BOLIVARIAN REPUBLIC OF	venezuela	venezuela	PAHO
	venezuela (bolivarian republic of)		
WALLIS AND FUTUNA	wallis and futuna islands	wallis and futuna	WPRO
