# Supplementary material for: The Availability and Consistency of Dengue Surveillance Data Provided Online by the World Health Organization
Source: PLoS Negl Trop Dis. 2015 Apr 14;9(4):e0003511. doi: 10.1371/journal.pntd.0003511 (PMC4397048; doi:10.1371/journal.pntd.0003511)
Supplement: S1 Fig — (A) Data for “all” cases (dengue fever and dengue hemorrhagic fever, DHF), (B) data for “all” deaths, and (C) data for DHF cases. Data availability from DengueNet is indicated in green, from WPRO in blue, from SEARO in yellow, and from PAHO in red. Data points that are available from both DengueNet and a regional office are shown in pink. (PDF) [file pntd.0003511.s002.pdf]

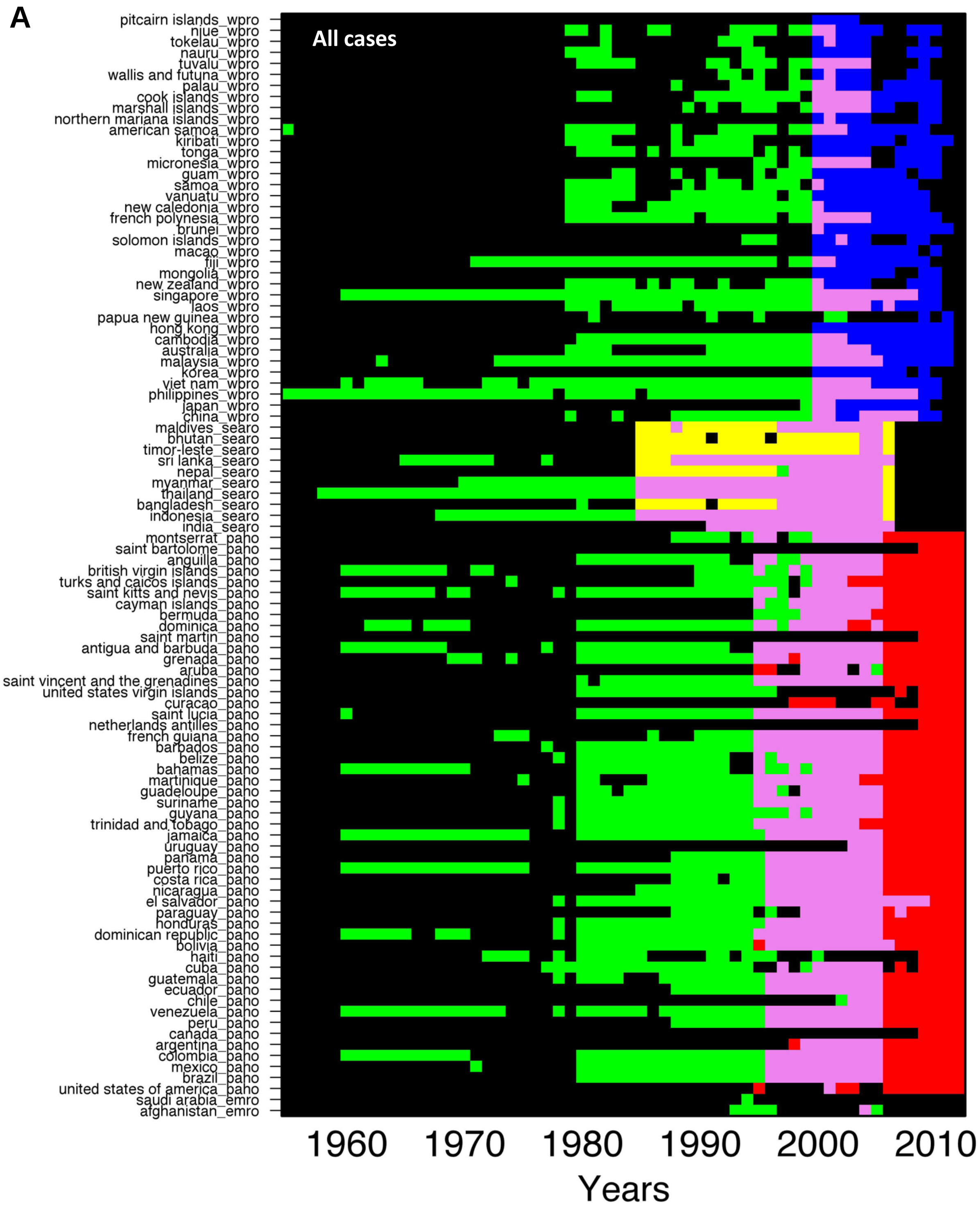

Figure S1. Data availability from WHO DengueNet and Regional Offices per country: 1955-2012. (A) Data for "all cases" (dengue fever and dengue hemorrhagic fever, DHF), **(B)** data for "all deaths", and **(C)** data for DHF cases. Data availability from DengueNet is indicated in green, from WPRO in blue, from SEARO in yellow, and from PAHO in red. Data points that are available from both DengueNet and a regional office are shown in pink.

B

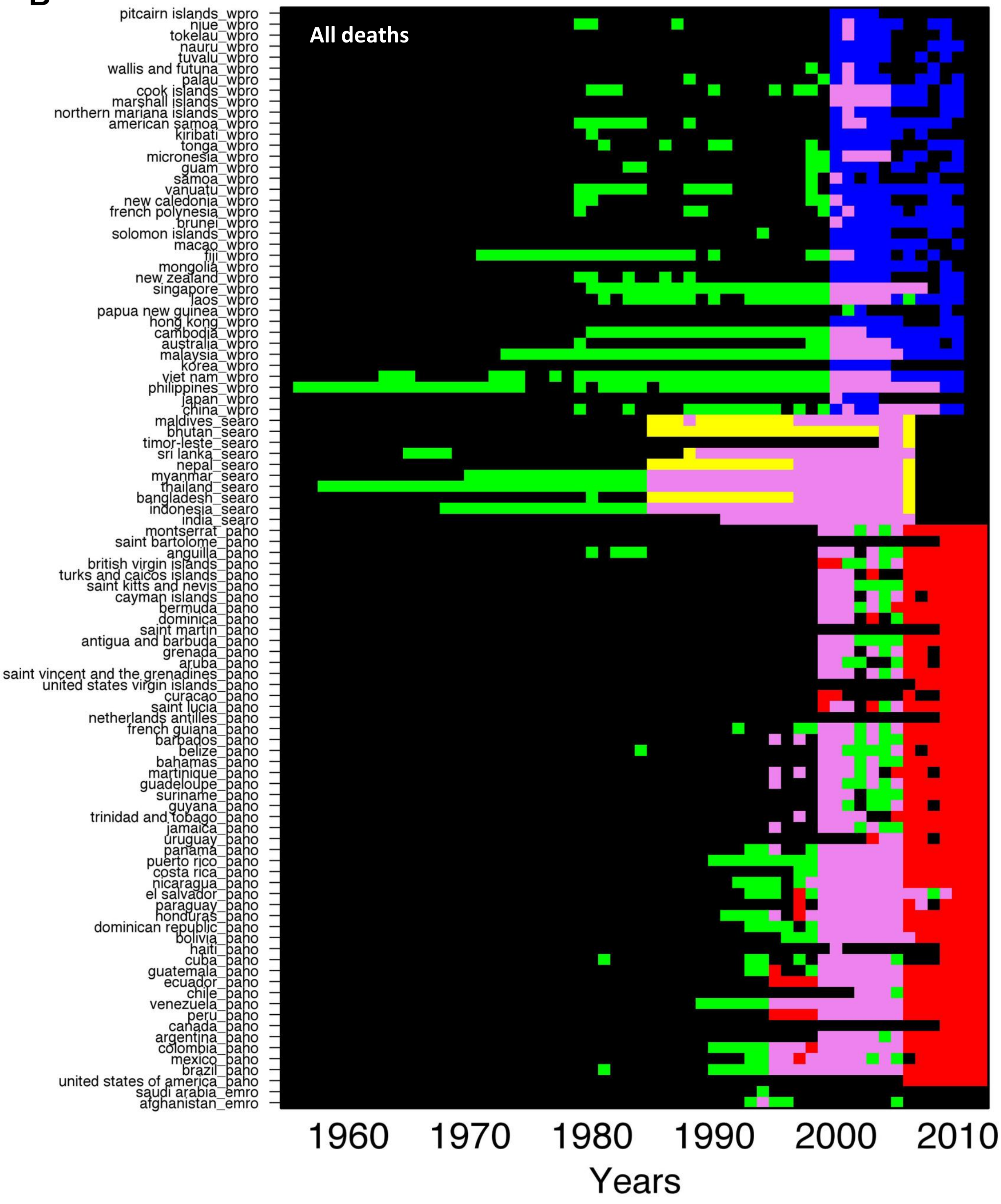

All deaths
pitcairn islands_wpro
niue_wpro
tokelau_wpro
nauru_wpro
tuvalu_wpro
wallis and futuna_wpro
palau_wpro
cook islands_wpro
marshall islands_wpro
northern mariana islands_wpro
american samoa_wpro
kiribati_wpro
tonga_wpro
micronesia_wpro
guam_wpro
samoa_wpro
vanuatu_wpro
new caledonia_wpro
french polynesia_wpro
brunei_wpro
solomon islands_wpro
macao_wpro
fiji_wpro
mongolia_wpro
new zealand_wpro
singapore_wpro
laos_wpro
papua new guinea_wpro
hong kong_wpro
cambodia_wpro
australia_wpro
malaysia_wpro
korea_wpro
viet nam_wpro
philippines_wpro
japan_wpro
china_wpro
maldives_searo
bhutan_searo
timor-leste_searo
sri lanka_searo
nepal_searo
myanmar_searo
thailand_searo
bangladesh_searo
indonesia_searo
india_searo
montserrat_paho
saint bartolome_paho
anguilla_paho
british virgin islands_paho
turks and caicos islands_paho
saint kitts and nevis_paho
cayman islands_paho
bermuda_paho
dominica_paho
saint martin_paho
antigua and barbuda_paho
grenada_paho
aruba_paho
saint vincent and the grenadines_paho
united states virgin islands_paho
curacao_paho
saint lucia_paho
netherlands antilles_paho
french guiana_paho
barbados_paho
belize_paho
bahamas_paho
martinique_paho
guadeloupe_paho
suriname_paho
guyana_paho
trinidad and tobago_paho
jamaica_paho
uruguay_paho
panama_paho
puerto rico_paho
costa rica_paho
nicaragua_paho
el salvador_paho
paraguay_paho
honduras_paho
dominican republic_paho
bolivia_paho
haiti_paho
cuba_paho
guatemala_paho
ecuador_paho
chile_paho
venezuela_paho
peru_paho
canada_paho
argentina_paho
colombia_paho
mexico_paho
brazil_paho
united states of america_paho
saudi arabia_emro
afghanistan_emro
1960
1970
1980
1990
2000
2010
Years

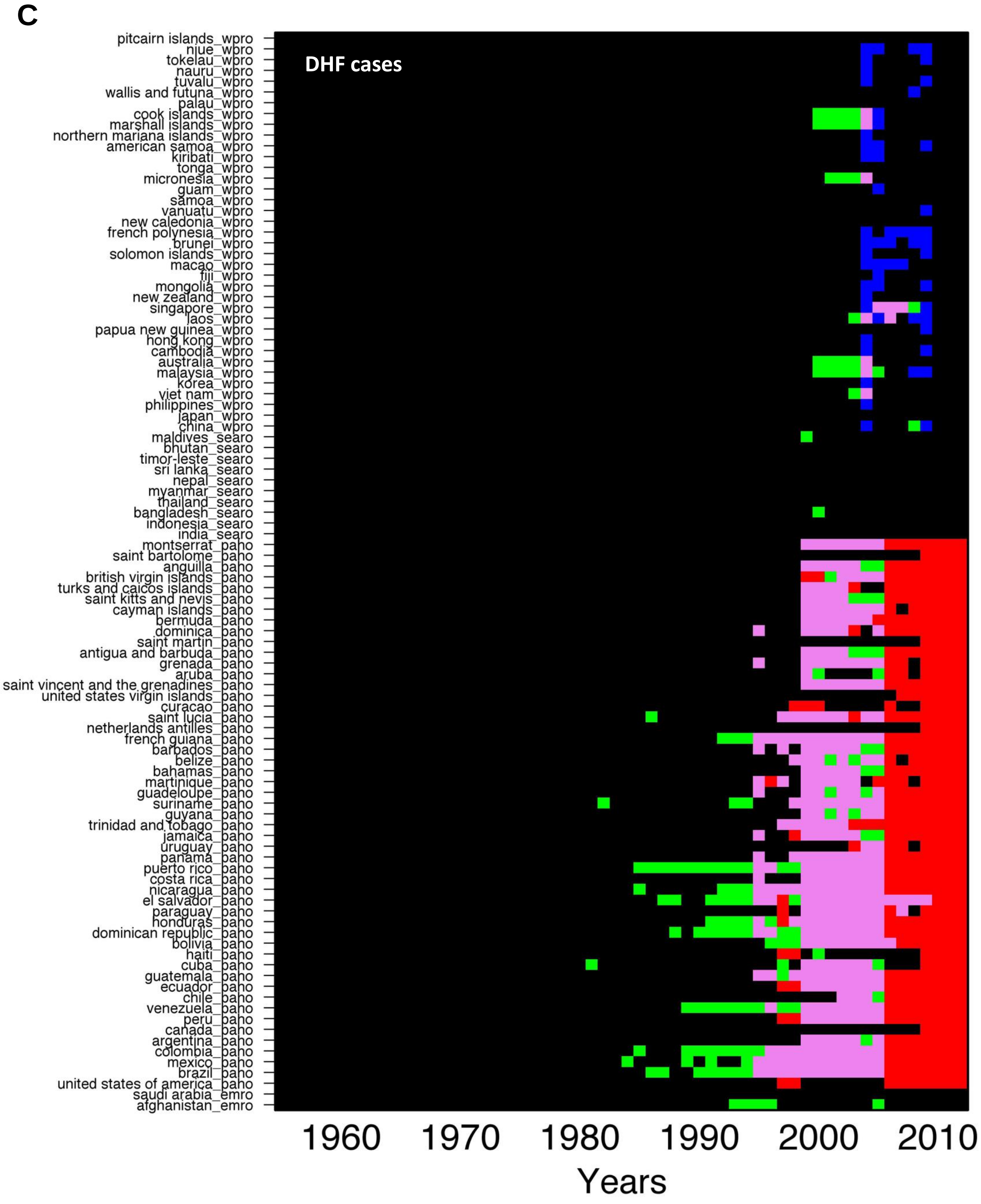
C
DHF cases
pitcairn islands_wpro
niue_wpro
tokelau_wpro
nauru_wpro
tuvalu_wpro
wallis and futuna_wpro
palau_wpro
cook islands_wpro
marshall islands_wpro
northern mariana islands_wpro
american samoa_wpro
kiribati_wpro
tonga_wpro
micronesia_wpro
guam_wpro
samoa_wpro
vanuatu_wpro
new caledonia_wpro
french polynesia_wpro
brunei_wpro
solomon islands_wpro
macao_wpro
fiji_wpro
mongolia_wpro
new zealand_wpro
singapore_wpro
laos_wpro
papua new guinea_wpro
hong kong_wpro
cambodia_wpro
australia_wpro
malaysia_wpro
korea_wpro
viet nam_wpro
philippines_wpro
japan_wpro
china_wpro
maldives_searo
bhutan_searo
timor-leste_searo
sri lanka_searo
nepal_searo
myanmar_searo
thailand_searo
bangladesh_searo
indonesia_searo
india_searo
montserrat_paho
saint bartolome_paho
anguilla_paho
british virgin islands_paho
turks and caicos islands_paho
saint kitts and nevis_paho
cayman islands_paho
bermuda_paho
dominica_paho
saint martin_paho
antigua and barbuda_paho
grenada_paho
aruba_paho
saint vincent and the grenadines_paho
united states virgin islands_paho
curacao_paho
saint lucia_paho
netherlands antilles_paho
french guiana_paho
barbados_paho
belize_paho
bahamas_paho
martinique_paho
guadeloupe_paho
suriname_paho
guyana_paho
trinidad and tobago_paho
jamaica_paho
uruguay_paho
panama_paho
puerto rico_paho
costa rica_paho
nicaragua_paho
el salvador_paho
paraguay_paho
honduras_paho
dominican republic_paho
bolivia_paho
haiti_paho
cuba_paho
guatemala_paho
ecuador_paho
chile_paho
venezuela_paho
peru_paho
canada_paho
argentina_paho
colombia_paho
mexico_paho
brazil_paho
united states of america_paho
saudi arabia_emro
afghanistan_emro
1960
1970
1980
1990
2000
2010
Years
