# Supplementary material for: The Availability and Consistency of Dengue Surveillance Data Provided Online by the World Health Organization
Source: PLoS Negl Trop Dis. 2015 Apr 14;9(4):e0003511. doi: 10.1371/journal.pntd.0003511 (PMC4397048; doi:10.1371/journal.pntd.0003511)
Supplement: S2 Fig — Data were available from both sources for years between 2000 and 2005 for (A) “all” cases, (B) “all deaths”, and (C) DHF cases. (PDF) [file pntd.0003511.s003.pdf]

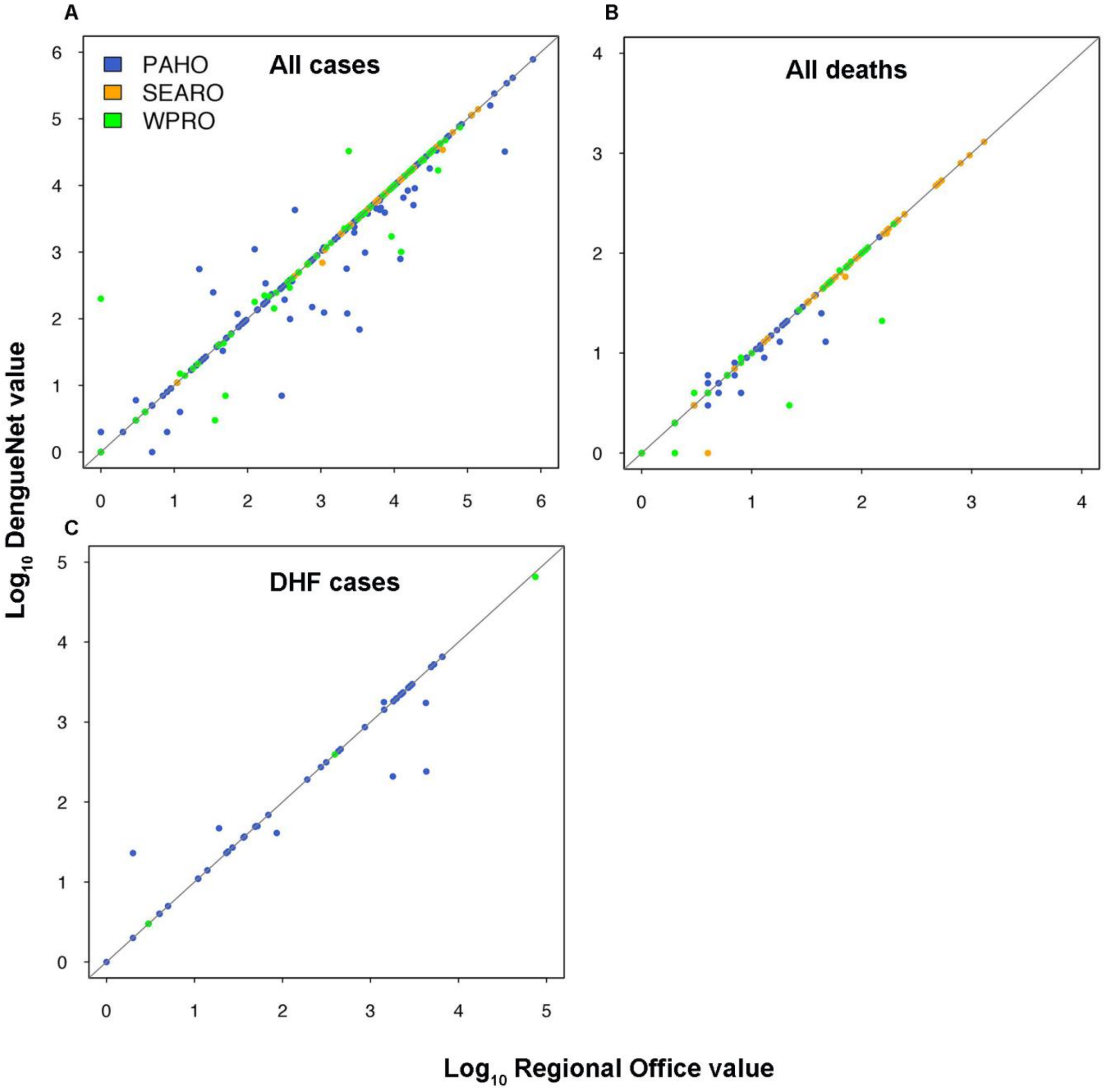

Figure S2. Consistency of data from DengueNet and WHO Regional Offices. Data were available from both sources for years between 2000 and 2005 for **(A)** "all cases", **(B)** "all deaths", and **(C)** DHF cases.
