# Supplementary material for: The Availability and Consistency of Dengue Surveillance Data Provided Online by the World Health Organization
Source: PLoS Negl Trop Dis. 2015 Apr 14;9(4):e0003511. doi: 10.1371/journal.pntd.0003511 (PMC4397048; doi:10.1371/journal.pntd.0003511)
Supplement: S3 Fig — Per year, the distribution across countries is shown of the number of calendar months (A) and the percent of all provinces (B) with data available for “all” cases. (PDF) [file pntd.0003511.s004.pdf]

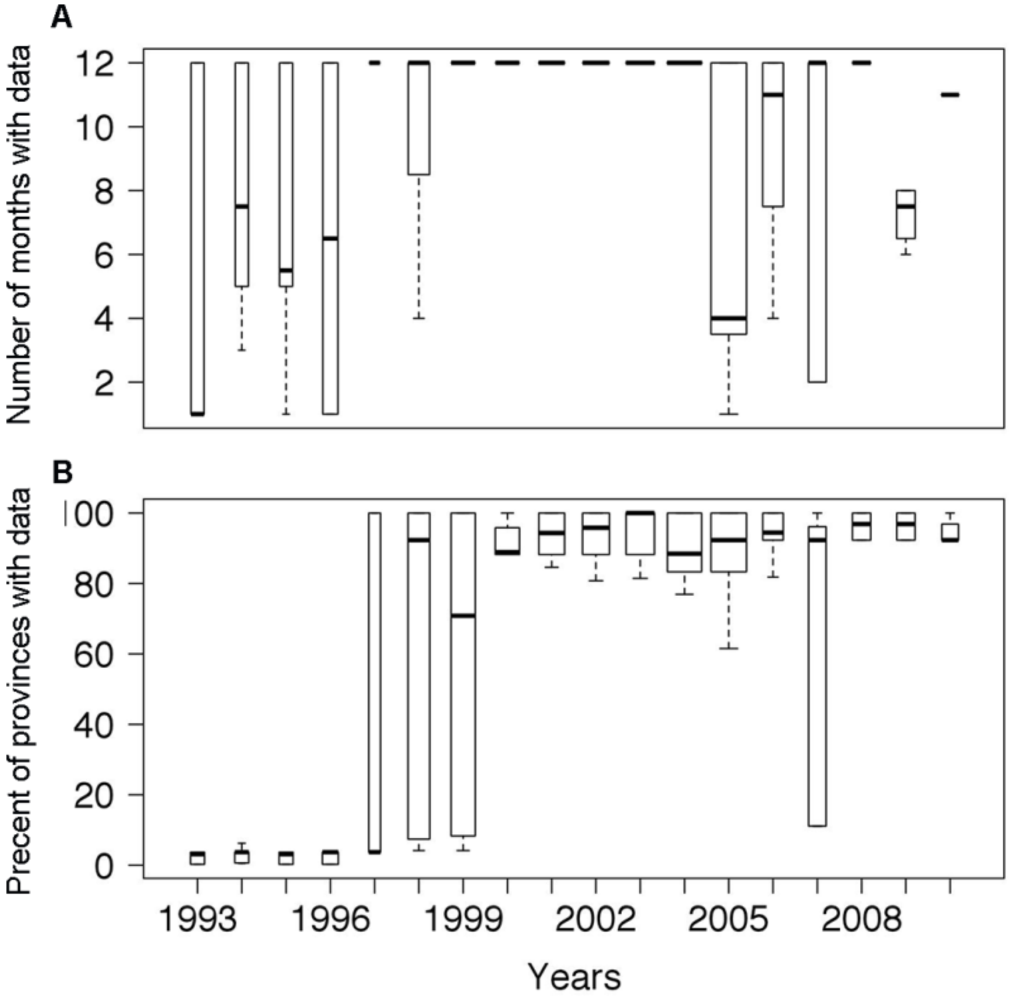

Figure S3. Completeness of DengueNet data on "all cases" at provincial/monthly resolution across countries by year. Per year, the distribution across countries is shown of the number of calendar months **(A)** and the percent of all provinces **(B)** with data available for "all cases".
